# Differences by transplant type in stool multiplex PCR testing for acute diarrhea in post-solid organ transplantation

**DOI:** 10.3389/fgstr.2022.1064187

**Published:** 2022-12-22

**Authors:** Abhishek Verma, Ashley M. Hine, Andrew Joelson, Rena Mei, Benjamin Lebwohl, Jordan E. Axelrad

**Affiliations:** ^1^ Department of Medicine, Yale New Haven Hospital, New Haven, CT, United States; ^2^ University of Connecticut School of Medicine, University of Connecticut, Farmington, CT, United States; ^3^ Division of Digestive and Liver Diseases, Department of Medicine, Columbia University Irving Medical Center, New York, NY, United States; ^4^ Department of Medicine, University of California, San Francisco, San Francisco, CA, United States; ^5^ Division of Gastroenterology, NYU Langone Health, Inflammatory Bowel Disease Center at NYU Langone Health, New York, NY, United States

**Keywords:** multiplex (RT)-PCR, diarrhea, solid organ transplantation, renal transplantation, lung transplantation, hospitalization, enteric infection

## Abstract

**Background:**

Diarrhea in solid organ transplant (SOT) recipients is common, morbid, and increasingly evaluated using multiplex gastrointestinal PCR panel (GI panel) testing. We aimed to characterize differences between transplant organ types in GI panel evaluation of acute diarrhea in SOT recipients.

**Methods:**

We performed a dual-center retrospective cross-sectional study of adult SOT recipients with acute diarrhea who underwent GI panel testing. Demographic, transplant, testing context, and GI panel data were collected. Patients were stratified by transplant type. The primary outcome was a positive GI panel.

**Results:**

Of 300 transplant recipients (58 heart, 65 liver, 68 lung, and 109 renal), 118 had a positive GI panel. Renal transplant status correlated with more frequently positive GI panel and less frequent hospitalization. In a multivariate analysis adjusting for demographic factors, hospitalization, immunosuppression, and transplant age, renal transplantation was independently associated with a positive GI panel compared to lung transplantation (aOR 2.98, 95% CI 1.27-7.16). Older transplant age and outpatient testing were also independently associated with a positive GI panel. The GI panel result was associated with changes to antibiotic management.

**Conclusions:**

In the evaluation of SOT recipients with acute diarrhea, GI panel result varies by transplant type, transplant age, and testing location and may affect subsequent antimicrobial therapy.

## Introduction

Diarrheal illness affects 20-50% of solid organ transplant (SOT) recipients (1–3) and carries significant associated morbidity. SOT recipients with diarrhea have higher rates of dehydration-associated medication toxicity, organ rejection, hospitalization, and death (4–7) Additionally, SOT patients with diarrhea experience worse quality of life compared to those without diarrhea (8).

Although infectious diarrhea is more common in SOT recipients compared to those without transplanted organs (9) non-infectious etiologies are also common in this population. These include medication toxicity (e.g. from mycophenolate mofetil), inflammatory bowel disease, post-transplant lymphoproliferative disease, and malignancy (10, 11) Polymerase chain reaction (PCR) stool testing in SOT recipients with diarrhea detects infectious pathogens at a higher rate compared to conventional stool testing (12) thus, the gastrointestinal stool PCR panel (GI panel) is a guideline-recommended tool for the evaluation of diarrhea in this population (10).

Current studies on the evaluation of this common and morbid condition in SOT recipients are limited by focusing on a single pathogen (e.g. *Clostridioides difficile*) or in studies evaluating the utility of the GI panel on a single transplant organ type. A comparison of the GI panel among transplant types has not been explored. We aimed to characterize differences between organ transplant types in the evaluation of acute diarrhea by GI panel in single solid organ transplant recipients, including any potential relationship to subsequent antimicrobial therapy.

## Methods

### Patient selection

We performed a cross-sectional study of the electronic medical records at two urban quaternary care institutions serving New York City (NYU Langone Health and NewYork-Presbyterian-Columbia University Medical Center). Adult patients with a history of single solid organ (lung, liver, kidney, or heart) transplant who underwent stool testing between 2016 and 2019 with the FilmArray GI pathogen panel (BioFire Diagnostics, Salt Lake City, UT) for acute diarrhea were included in the study. Transplant status was evaluated using International Classification of Disease-10 coding (13) and confirmed by manual chart review. Acute diarrhea was ascertained by provider chart documentation description and defined as 3 or more unformed stools in 24 hours for less than 14 days. Patients with more than one transplanted organ or diarrheal illness for longer than 14 days were excluded from the analyses.

### Variables and definitions

The following demographic data were collected: age, sex, race, ethnicity, Charlson’s comorbidity index (14) and HIV status. Data on testing context including travel 30 days prior to testing, recent antibiotics or hospitalization, test setting (outpatient, emergency department [ED], inpatient), symptoms at testing, and sexual exposure were also collected. Transplant data collected included organ type, transplant age, number and class of any immunosuppression medications, and use of antimicrobial prophylaxis against opportunistic infections (OI) at the time of testing.

Data on antibiotic therapy was also recorded. Those who received antibiotics were divided into four groups: (1) subjects who received an incomplete course of empiric antibiotics that was discontinued after GI panel result, (2) subjects who had empiric antibiotic therapy started before GI panel result that was narrowed to directed therapy after GI panel result, (3) subjects with antibiotic therapy initiation only after GI panel result, and (4) subjects who received a full empiric course of antibiotics that was unchanged after GI panel result. The first three groups were considered subjects who had antibiotic management changes based on GI panel result.

Only the first GI panel tested for diarrheal symptoms was included for each subject during this time span. Enteric infection was defined as a positive result on the GI panel. The BioFire FilmArray GI Panel can identify the nucleic acids of 23 pathogens (14 bacteria, 5 viruses, and 4 parasites) within one hour, and is performed on stool samples stored in Cary Blair transport medium. The test has a clinical sensitivity and specificity of 94.5% and 100% (15, 16) The pathogens are: *Campylobacter jejuni*, *Clostridioides difficile* (toxin A/B), *Plesiomonas shigelloides*, *Salmonella* species, *Yersinia enterocolitica*, *Vibrio parahaemolyticus*, *Vibrio vulnificus*, *Vibrio cholerae*, enteroaggregative *Escherichia coli* (EAEC), enteropathogenic *E. coli* (EPEC), enterotoxigenic *E. coli* (ETEC) lt/st, Shiga-like toxin-producing *E. coli* (STEC) stx1/stx2, *E. coli* O157, *Shigella*/enteroinvasive *E. coli* (EIEC), adenovirus F40/41, astrovirus, norovirus GI//GII, rotavirus A, sapovirus (I, II, IV, and V), *Cryptosporidium* species, *Cyclospora cayatenesis*, *Entamoeba histolytica*, and *Giardia lamblia*. There were no institutional limitations on ordering stool PCR testing. Serologic, endoscopic, and histologic chart data were used where available to identify other infectious diarrheal etiologies such as CMV and *Mycobacterium avium* complex (MAC) at one study center (NYU).

### Outcome and statistical analyses

The primary outcome was the presence of enteric infection by stool GI panel. Secondary outcomes included the following: ED visit, surgery, death, endoscopy, or hospitalization within 30 days of stool testing. If patients were hospitalized at the time of testing, only re-hospitalization was considered a 30-day hospitalization event.

Patients were stratified by transplant organ type. Categorical variables were analyzed using chi-squared testing, and continuous variables were analyzed using the ANOVA test and reported with the median and interquartile range values. A logistic multivariable regression analysis was then performed with GI panel result as the primary outcome. Certain variables were included *a priori*: age, sex, race, Charlson’s comorbidity index, use of OI prophylaxis, transplant type, and transplant age. Variables demonstrating statistical significance in the univariate analysis were added to the *a priori* variables in the multivariate analysis. All statistical analyses were conducted using R version 3.3.3 (17) A P value <0.05 was considered statistically significant. The study was approved by the New York University Institutional Review Board [NYU IRB s18-01121 approval date 8/26/2018].

## Results

### Baseline characteristics

Of 302 solid organ transplant patients evaluated with the GI panel from 2016 and 2019 for acute diarrhea, 300 had a single transplanted organ and were included in the study (**Table 1**). Of all subjects, 131 (43.7%) were female. The median age at testing was 59.4 years (interquartile range [IQR] 44.1-67.1 years). There were 58 (19%) heart, 65 (22%) liver, 68 (23%) lung, and 109 (36%) renal transplant patients. Sixty-eight percent were inpatients at the time of testing. Demographics for each transplant type are outlined in **Table 1**.

**Table 1:** Baseline Characteristics by Transplant Type.

	Evaluation by Transplant Type					
	Heart Transplant (n=58)	Liver Transplant (n=65)	Lung Transplant (n=68)	Renal Transplant (n=109)	P-Value	
Age (years), median (IQR)	59.7 (40.1-68.0)	61.0 (39.0-66.3)	61.7 (53.7-69.0)	53.9 (42.0-65.0)	0.161	
Female	22 (37.9)	22 (33.8)	37 (54.4)	50 (45.9)	0.081	
Race					<0.001	
White	31 (53.4)	37 (56.9)	52 (76.5)	27 (24.8)		
Non-White	27 (46.6)	28 (43.1)	16 (23.5)	82 (75.2)		
Ethnicity					0.097	
Non-Hispanic	36 (62.1)	42 (64.6)	56 (82.4)	67 (61.5)		
Hispanic	11 (19.0)	10 (15.4)	8 (11.8)	21 (19.3)		
Other/Declined/Unknown	11 (19.0)	13 (0.20)	4 (5.9)	21 (19.3)		
Charlson’s Comorbidity Index, median (IQR)	4 (3-5)	4 (3-6)	3 (2.75-5)	4 (3-6)	0.007	
Human Immunodeficiency Virus (HIV), n (%)	0 (0)	3 (4.6)	0 (0)	3 (2.8)	0.161	
Sexual Exposure, n (%)	0 (0)	10 (15.4)	0 (0)	2 (1.8)	0.526	
Duration of Transplant, median (IQR)	2392 (612-5270)	1280 (245-2806)	643 (120-2404)	711 (162-2364)	0.004	
Transplant Age, n (%)					0.153	
0-3 months	0 (0)	8 (12.3)	7 (10.3)	11 (10.1)		
-12 months	7 (12.1)	7 (10.8)	12 (17.6)	18 (16.5)		
12 months or older	51 (87.9)	50 (76.9)	49 (72.1)	80 (73.4)		
Immunosuppression, n (%)						
None	1 (1.7)	1 (1.5)	0 (0)	4 (3.7)	0.387	
Calcineurin Inhibitor	56 (96.6)	56 (86.2)	66 (97.1)	97 (89.0)	0.046	
Mycophenolate Mofetil/Azathioprine	34 (58.6)	37 (56.9)	56 (82.4)	91 (83.5)	<0.001	
Steroids	45 (77.6)	31 (47.7)	67 (98.5)	78 (71.6)	<0.001	
mTOR Inhibitor	9 (15.6)	9 (13.8)	1 (1.5)	2 (1.8)	<0.001	
Other	0 (0)	3 (4.6)	4 (5.9)	5 (4.6)	0.361	
Immunosuppression Held, n (%)	13 (23.2)	6 (9.2)	8 (11.8)	21 (19.3)	0.124	
Number of Immunosuppressive Agents, n (%)					<0.001	
0	1 (1.7)	1 (1.5)	0 (0)	4 (3.7)		
1	2 (3.4)	18 (27.7)	0 (0)	6 (5.5)		
2	25 (43.1)	21 (32.3)	13 (19.1)	32 (29.4)		
3	28 (48.3)	24 (36.9)	52 (76.5)	65 (59.6)		
4	2 (3.4)	1 (1.5)	3 (4.4)	2 (1.8)		
On Opportunistic Infection Prophylaxis	18 (31.0)	20 (30.8)	54 (79.4)	51 (46.8)	<0.001	
Travel 30 Days Prior to PCR	4 (6.9)	3 (4.6)	1 (1.5)	5 (4.6)	0.514	
Recent Antibiotics or Hospitalization	30 (51.7)	33 (50.8)	50 (73.5)	53 (48.6)	0.009	
Place of PCR					0.012	
Outpatient	15 (25.9)	16 (24.6)	14 (20.6)	37 (33.9)		
Inpatient	43 (74.1)	46 (70.8)	54 (79.4)	63 (57.8)		
Emergency Department	0 (0)	3 (4.6)	0 (0)	9 (8.3)		
Symptoms at PCR						
Hematochezia	0 (0)	0 (0)	1 (1.5)	3 (2.8)	0.342	
Abdominal Pain	13 (22.4)	20 (30.8)	14 (20.6)	24 (22.0)	0.496	
Fever	11 (19.0)	15 (23.1)	14 (20.6)	26 (23.9)	0.884	
Nausea/Vomiting	11 (19.0)	10 (15.4)	12 (17.6)	31 (28.4)	0.145	
Other/Unknown	1 (1.7)	2 (3.1)	8 (11.8)	0 (0)	<0.001	
Hospitalization	44 (75.9)	44 (67.7)	53 (77.9)	63 (57.8)	0.019
Length of Stay (days), median (IQR)	6 (2-11)	9 (4-18)	8.5 (6-24.3)	6 (4-10)	0.006

### Outcomes

A positive GI panel was present in 118 (39.3%) patients: 55 with viral pathogens, 70 with bacterial pathogens, 6 with parasitic pathogens, and 23 with multiple pathogen types (**Table 2**). *Clostridioides difficile* was the most common pathogen with 48 (41%) infected subjects, followed by norovirus in 41 (35%) subjects, and EPEC in 32 (27%) subjects. Nine of the patients undergoing GI panel testing were also diagnosed with cytomegalovirus (CMV) infection; seven of these nine were renal transplant recipients and 5 had a concomitant positive GI panel. Of 31 patients with a negative GI panel at one center (NYU), 2 were diagnosed with CMV. No other pathogen was identified as the cause of diarrhea in the remaining patients (**Supplementary Table 1**).

**Table 2:** GI panel results by transplant type.

			Evaluation By Transplant Type							
		Heart Transplant (n=58)	Liver Transplant (n=65)			Lung Transplant (n=68)	Renal Transplant (n=109)			P-Value
Positive GI PCR Panel		24 (41.4)	18 (27.7)			17 (25.0)	59 (54.1)			<0.001
Viral infection, n (%)		12 (20.7)	6 (9.2)			11 (16.2)	26 (23.9)			0.100
Bacterial Infection, n (%)		13 (22.4)	14 (21.5)			6 (8.8)	37 (33.9)			0.002
Parasitic Infection, n (%)		0 (0)	0 (0)			2 (2.9)	4 (3.7)			0.224
Multiple Pathogens		6 (10.3)	5 (7.7)			2 (2.9)	10 (9.2)			0.588
Viral Infection										
Adenovirus	1 (1.7)			0 (0)	0 (0)			0 (0)	0.242	
Astrovirus	0 (0)			0 (0)	0 (0)			1 (0.92)	0.624	
Norovirus	8 (13.8)			5 (7.7)	8 (11.8)			20 (18.3)	0.241	
Rotavirus	1 (1.7)			1 (1.5)	2 (2.9)			0 (0)	0.408	
Sapovirus	2 (3.4)			1 (1.5)	2 (2.9)			4 (3.7)	0.877	
Bacterial Infection										
*Campylobacter* species	2 (3.4)			0 (0)	0 (0)			4 (3.7)	0.180	
*Clostridioides difficile*	10 (17.2)			8 (12.3)	10 (14.7)			20 (18.3)	0.739	
*Plesiomonas shigelloides*	NA			NA	NA			NA	NA	
*Salmonella* species	NA			NA	NA			NA	NA	
*Yersinia enterocolitica*	0 (0)			2 (3.1)	0 (0)			0 (0)	0.064	
*Vibrio parahaemolyticus*	0 (0)			0 (0)	1 (1.5)			1 (0.92)	0.661	
*Vibrio vulnificus*	NA			NA	NA			NA	NA	
*Vibrio cholerae*	NA			NA	NA			NA	NA	
*Enteroaggregative E. coli (EAEC)*	2 (3.4)			4 (6.2)	1 (1.5)			5 (4.6)	0.560	
*Enteropathogenic E. coli (EPEC)*	8 (13.8)			7 (10.8)	0 (0)			17 (15.6)	0.009	
*Enterotoxigenic E. coli (ETEC)*	2 (3.4)			0 (0)	0 (0)			1 (0.92)	0.182	
*Shiga-like Toxin-producing E. coli (STEC)*	0 (0)			1 (1.5)	0 (0)			2 (1.8)	0.526	
*E. coli O157*	NA			NA	NA			NA	NA	
*Shigella/Enteroinvasive E. coli (EIEC)*	0 (0)			1 (1.5)	0 (0)			0 (0)	0.305	
Parasitic Infection										
*Cryptosporidium*	0 (0)			1 (1.5)	0 (0)			2 (1.8)	0.526	
*Cyclospora cayatenesis*	NA			NA	NA			NA	NA	
*Entamoeba histolytica*	NA			NA	NA			NA	NA	
*Giardia lamblia*	0 (0)			0 (0)	3 (4.4)			2 (1.8)	0.156	
Antibiotics Prescribed	23 (39.7)			29 (44.6)	15 (22.1)			60 (55.0)	<0.001	
Incomplete Empiric Course Stopped After GI Panel Result	2 (3.4)			1 (1.5)	1 (1.5)			3 (2.8)	0.849	
Empiric Antibiotics Narrowed After GI Panel Result	6 (10.3)			1 (1.5)	1 (1.5)			8 (7.3)	0.055	
Directed Antibiotics Initiated After GI Panel Result	10 (17.2)			7 (10.8)	7 (10.3)			33 (30.3)	0.002	
Full Empiric Course Unchanged After GI Panel Result	5 (8.6)			20 (30.8)	6 (8.8)			16 (14.7)	0.001	
Antibiotic Management Affected by GI Panel Result	18/23 (78.2)		9/29 (31.0)			9/15 (60.0)		44/60 (73.3)		<0.001
Hospitalization Within 30 Days of PCR	8 (13.8)		10 (15.4)			10 (14.7)		21 (19.3)		0.769
ER Visit Within 30 Days of PCR	2 (3.4)		3 (4.6)			1 (1.5)		4 (3.7)		0.776
Surgery Within 30 Days of PCR	1 (1.7)		3 (4.6)			7 (10.3)		7 (6.4)		0.225
Death Within 30 Days of PCR	9 (15.5)		9 (13.8)			12 (17.6)		8 (7.3)		0.185
Endoscopy Within 30 Days of PCR	4 (6.9)		15 (23.1)			2 (2.9)		11 (10.1)		0.001

NA, Not Available.

Within 30 days after stool testing, 38 (12.7%) patients died, 32 (10.7%) underwent endoscopy (18), (6%) underwent surgery, and 49 (16.3%) were subsequently either hospitalized or re-hospitalized. There was no correlation between a positive GI panel and 30-day mortality (p=0.185).

### Comparison among transplant types

**Figure 1:**
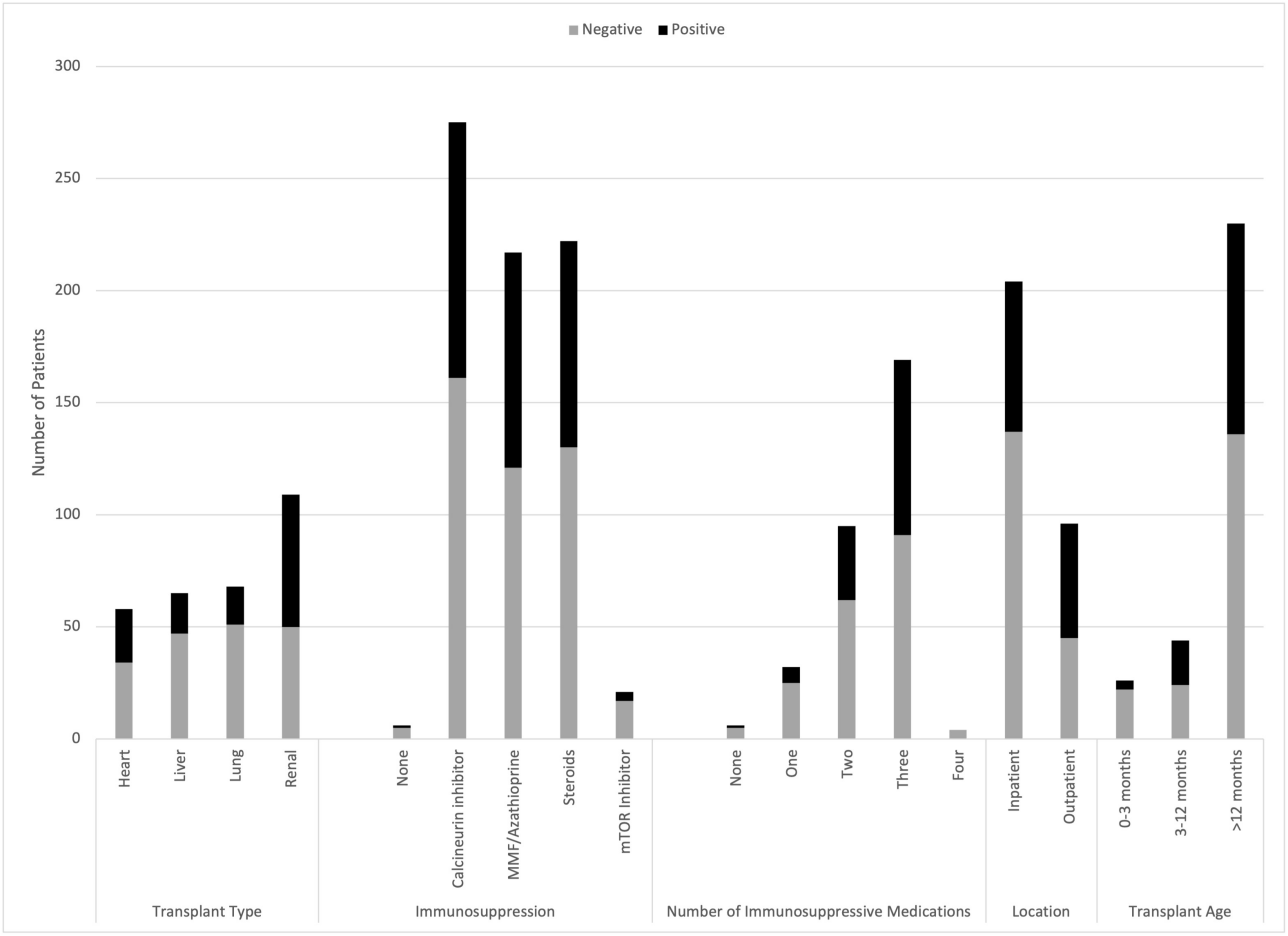
Prevalence of Infectious Diarrhea Varies Based on Transplant Type, Immunosuppression, Location, and Transplant Age.

A positive GI panel was more common in renal transplant recipients (54% renal vs. 41% heart vs. 28% liver vs. 25% lung, p<0.001; **Figure 1**; **Table 1**). Bacterial infection was less common in lung transplant recipients (8.8% lung vs. 34% renal vs. 22% heart vs. 22% liver, p=0.002; **Table 2**).

Renal transplant patients were more likely to be tested in the outpatient setting, and less likely to be hospitalized at testing for acute diarrhea (58% renal vs. 68% liver vs. 76% heart vs. 78% lung, p=0.019). Lung transplant patients were more likely to be prescribed OI prophylaxis, recently hospitalized, or have recently been prescribed antibiotics (**Table 1**). Lung and renal transplant patients were more likely to have a younger transplant age while heart and liver transplant patients were more likely to have an older transplant age (**Table 1**).

Immunosuppression class at the time of testing varied by transplant type (**Table 1**). There was notably a higher use of corticosteroids in lung transplant recipients compared to other groups (99% lung vs. 78% heart vs. 72% renal vs. 48% liver, p<0.001). The use of mTOR inhibitors was higher in heart and liver recipients compared to lung and renal recipients. The use of three immunosuppression agents was more common in lung transplant recipients, while the use of one was more common in liver recipients (**Table 1**). Liver transplant recipients were more likely to undergo endoscopy within 30 days of testing; 30-day outcomes were otherwise comparable among groups.

### Predictors of a positive GI panel

**Figure 2:**
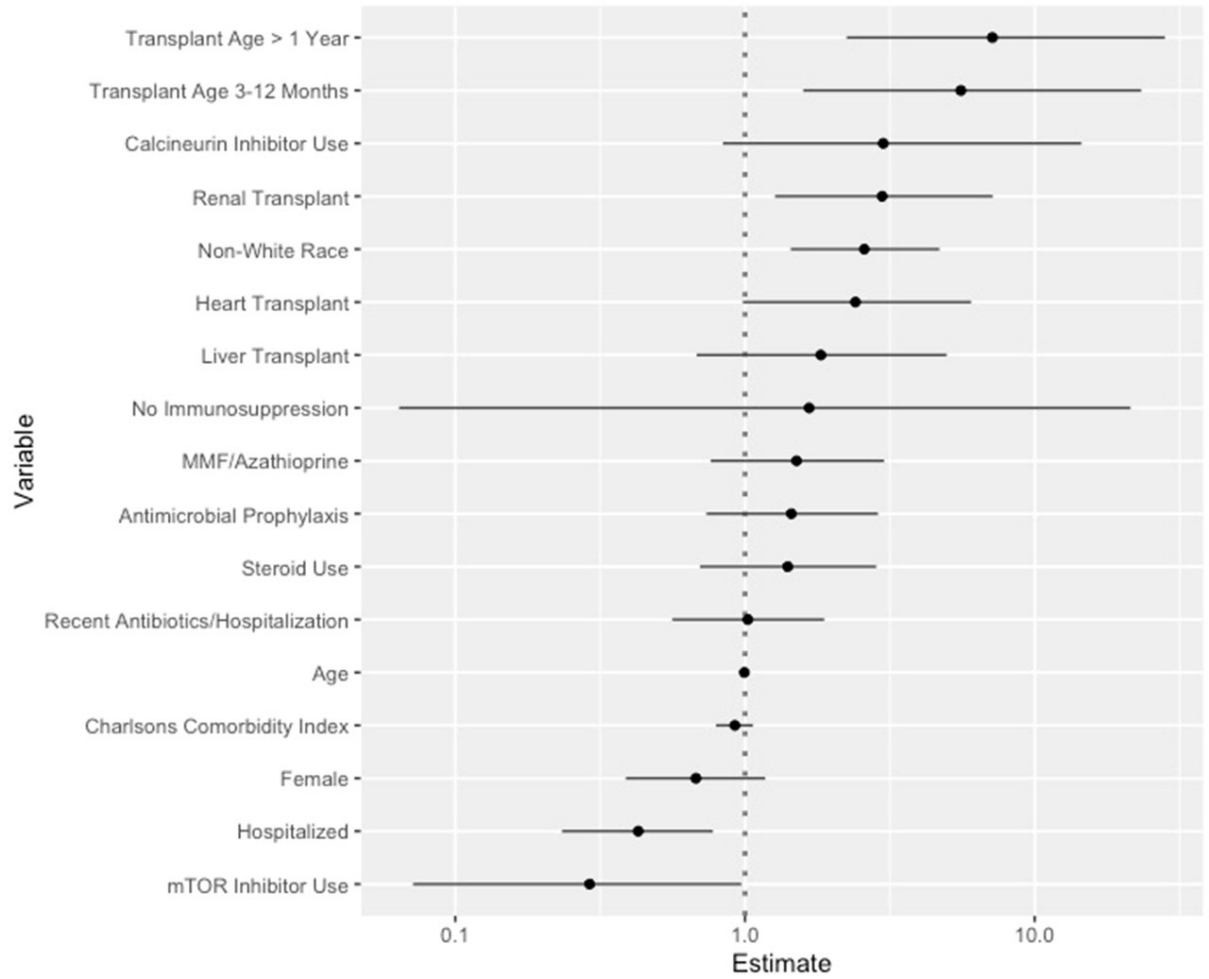
Multivariate Analyses of Factors Predictive of Positive GI Panel in Solid Organ Transplant Recipients with Acute Diarrhea. Coefficient plot of the multivariate logistic regression predicting positive GI panel with 95% confidence intervals demonstrated. Reference groups are as follows for the listed variables: transplant type: lung, transplant age: 0-3 months.

Given their distinct performance in the univariate analyses, the following 3 variables were added to the *a priori* variables in the multivariable analysis: hospitalization at testing, recent antibiotic use or hospitalization, and immunosuppression class. After adjusting for these variables, renal transplant status was independently associated with a positive GI panel compared to lung transplant status (adjusted odds ratio [aOR] 2.98, 95% confidence interval [CI] 1.27-7.16; **Figure 2**; **Supplementary Table 2**). Older transplant age and outpatient illness were also independently associated with a positive GI panel (**Figure 2**; **Supplementary Table 2**). The use of mTOR inhibitors was associated with a negative GI panel.

### Antibiotic therapy

Antibiotics were prescribed to 127 (42%) subjects (**Table 2**), of whom 80 (63%) had antibiotic therapy changed after GI panel result. Overall, antibiotic management changed more often in those with a positive GI panel (71 [60%] positive vs. 9 [4.9%] negative; p<0.001). Specifically, patients with a positive GI panel had their antibiotic therapy narrowed more often after GI panel result (14 [12%] positive vs. 2 [1.1%] negative; p<0.001) and were less likely to receive a full empiric course of antibiotic therapy (12 [10%] positive vs. 35 [19%] negative; p=0.035; **Supplementary Table 3**). Additionally, patients with a positive GI panel were started on directed antibiotic therapy after GI panel result more often (55 [47%] positive vs. 2 [1.1%] negative; p<0.001). GI panel result did not correlate with rates of discontinuation of empiric antibiotic therapy after availability of the result.

In comparing antibiotic therapy among transplant types, renal transplant recipients were more likely to receive antibiotics, especially directed antibiotic therapy initiated after GI panel result (30% renal vs. 10% lung vs. 17% heart vs. 11% liver; p=0.002; **Table 2**). Antibiotic management changed after GI panel result less often in patients with liver transplantation (31% liver vs. 60% lung vs. 73% renal vs. 78% heart; p<0.001; **Table 2**). There were no differences among transplant types in antibiotic de-escalation (discontinuation or narrowing of therapy) after GI panel result (**Table 2**).

### Location of testing

Due to the association between location of testing (i.e. inpatient vs. outpatient) and GI panel result, separate analyses were performed stratifying subjects by location type. In a comparison of hospitalized and non-hospitalized patients, hospitalized patients were less likely to have a pathogen identified on the GI panel (33% hospitalized vs. 53% non-hospitalized, p<0.001) and were more likely to have lung transplant status (26% hospitalized vs. 16% non-hospitalized, p=0.046). Hospitalized patients were less likely to have renal transplant status (31% hospitalized vs. 48% non-hospitalized, p=0.004). There was no association between immunosuppression type and hospitalization status. Fever at the time of evaluation and recent hospitalization or antibiotic use were more common in hospitalized subjects, and there was a higher 30-day mortality in hospitalized subjects. In a multivariable regression controlling for age, sex, race, transplant type, immunosuppression use, nausea/vomiting at testing, fever at testing, recent antibiotic use or hospitalization, Charlson’s comorbidity index, OI prophylaxis, and transplant age, there was an association between outpatient status and positive GI panel (OR 2.30, 95% CI 1.24-4.33), which did not persist when restricting the inpatient group to patients tested within 72 hours of hospitalization.

Separately, those tested in the outpatient setting were compared by transplant type (**Supplementary Tables 4 and 5**). The type and number of immunosuppression agents varied by transplant type (**Supplementary Table 4**). Lung transplant patients tested in the outpatient setting were more likely to be on OI prophylaxis (80% lung vs. 50% renal vs. 29% liver vs. 21% heart; p=0.004; **Supplementary Table 4**). There was no difference in GI panel result or 30-day outcomes among transplant types in the outpatient setting (**Supplementary Table 5**).

## Discussion

In this study of SOT recipients with acute diarrhea, GI panel results varied by transplant type. Specifically, compared to lung transplant recipients, renal transplant patients were more likely to have a positive GI panel even after adjusting for transplant age, immunosuppression, hospitalization, and use of OI prophylaxis. Lung transplant patients were more likely to be hospitalized, on OI prophylaxis, and on more intensive immunosuppression. Older transplant age and outpatient testing were also independently associated with a positive GI panel. The GI panel result was associated with changes to antibiotic therapy management.

Common infectious pathogens in SOT recipients with acute diarrhea include *Clostridioides difficile*, CMV, *Cryptosporidum*, EPEC, *Campylobacter*, and norovirus (9, 10, 12) Our study had a similar prevalence of pathogens with *C. difficile*, norovirus, and EPEC being the most commonly identified organisms, although only 9 subjects had confirmed CMV.

In this study, renal transplant status was independently associated with a positive GI panel compared to lung transplant status. In prior studies of *C. difficile* infection in SOT recipients, infection rates have been found to be both lower and similar in renal transplant patients compared to other transplant types (19, 20) However, such studies only focused on a single pathogen, and cannot be easily compared to a study evaluating infections from multiple various pathogens, each with its unique epidemiology. It is possible that the difference in GI panel result was related to more hospitalization and intensive immunosuppression seen in lung transplant recipients, as those factors would elevate the risk of non-infectious diarrhea or of infections with agents not evaluated for by the GI panel. However, the association between renal transplant status and positive GI panel persisted even after adjusting for immunosuppression, hospitalization, OI prophylaxis, and transplant age. Additionally, of the lung transplant recipients with a negative GI panel at one center, there were no alternative infectious etiologies identified, although this was limited by diagnostic investigations done at the discretion of providers.

It is important to note that this study was designed to identify differences in GI panel result among transplant types, and not to confirm whether differences in GI panel result represented differences in true enteric infection. While some data from the current study may suggest that true enteric infection is indeed more common in renal transplant recipients, the question of whether any difference in GI panel result among transplant type is due to true differences in infection susceptibility needs further investigation.

Older transplant age was independently associated with a positive GI panel. We selected the cutoffs of 3 months and 12 months as they are guideline-recommended times of immunosuppression de-escalation (21, 22) This finding is consistent with a prior study evaluating *C. difficile* infections in multiple transplant types; most cases occurred beyond 3 months of transplantation (20) It has been postulated that the increase in late-onset infectious diarrhea may be due to intensified immunosuppression from graft rejection or repeated antimicrobial exposure post-transplant (20) Our data does not seem to support either of these two hypotheses: the association between transplant age and positive GI panel in this study persisted even after controlling for immunosuppression and recent antibiotic use or hospitalization, suggesting against graft rejection-related or antimicrobial exposure-related etiologies of this association. However, we did not specifically collect graft rejection data, and the potential role of graft rejection in this association should be investigated further.

It is unlikely that pathogens not identified on the GI panel, such as CMV, would be responsible for the association between older transplant age and a positive GI panel. While immunosuppression is most significant in the immediate post-transplant period, patients are also more likely to be on CMV prophylaxis at this time and thus theoretically protected from CMV infection. This is supported by our finding that of 9 patients with CMV at one center in this study, only one was identified within the first 3 months after transplantation. Alternatively, the association between transplant age and GI panel positivity could simply be a result of higher patient vigilance against infection risk in the early post-operative period, which may wane with time. Further investigation of the relationship between time from transplant and positive GI panel is needed.

Of those subjects who received antibiotics in the study, the majority experienced changes to antibiotic therapy after the GI panel result was available; a positive GI panel correlated with more frequent narrowing of antibiotic therapy. Interestingly, a negative GI panel correlated with higher rates of a full empiric course of antibiotic therapy rather than discontinuation of empiric treatment after result availability as might be expected. This suggests that if the index of suspicion for infection is high enough a negative GI panel may not, in practice, influence the decision to discontinue antibiotic therapy in this patient population. A positive GI panel appears beneficial in targeting antibiotic therapy and improving antibiotic stewardship.

There was a strong inverse correlation between hospitalization and positive GI panel in this study. This is comparable to previous findings by Echenique, et al. in a 2015 study utilizing conventional stool testing, where hospitalized patients had a lower rate of identification of infectious pathogens as compared to non-hospitalized patients (5) One possible reason for this inverse association is that the GI panel is unable to identify infectious diarrhea from certain pathogens that may predispose to severe illness requiring hospitalization (e.g. CMV, MAC). This is potentially less likely, as patients with a negative GI panel at one center of the study were rarely diagnosed with such pathogens. At the same time, not every patient with a negative GI panel and no identifiable diarrheal etiology underwent endoscopic evaluation, which can identify abnormalities such as CMV colitis, drug toxicity, and IBD in 20-45% of patients (23, 24).

A second and more likely possible etiology of the inverse association between hospitalization and GI panel result could be that hospitalized patients are more prone to non-infectious causes of diarrhea, such as systemic illness, antibiotic-associated diarrhea or a bowel regimen while hospitalized. This is more likely given that the strong inverse association between hospitalization and GI panel result did not persist when restricting to inpatients tested within 72 hours of hospitalization. As previously studied, GI PCR testing beyond 72 hours of hospitalization has a low yield (25) Thus, while it may be that severe enough diarrhea may not be due to common infections, we cannot rule out the possibility of reverse causality, in which events early in the hospitalization lead to acute diarrhea.

Our study was limited first by its retrospective nature; we did not have insight into the clinical context informing the decision to obtain a GI panel, alternative non-infectious diagnoses in those with a negative GI panel, nor subsequent clinical response to antibiotic therapy. We additionally did not stipulate serologic or endoscopic testing for CMV as part of our study population definition. This could lead to the under-detection of a major and common cause of diarrhea in this population (10) However, of the 9 patients with a CMV diagnosis, the vast majority were renal transplant patients, and more than half had a positive GI panel. Thus, it is less likely that this would have ultimately changed the associations revealed by this study.

The GI panel may not reliably distinguish between infected and colonized subjects, as the isolation of genetic material *via* PCR testing is not necessarily equivalent to the presence of viable organisms in the alimentary tract; accordingly, our findings may not be entirely extrapolated to differences in true enteric infection, especially given the absence of quantitative PCR data for positive testing in our study. Rather, our results should be interpreted as the utility of GI panel testing in this population. We additionally did not record graft rejection data in our study, and thus were unable to relate the identified risk factors to graft outcomes.

In conclusion, GI panel results differ by transplant type when used to evaluate acute diarrhea in SOT patients. Renal transplant patients compared to lung transplant patients are more likely to be tested in the outpatient setting and to have a positive GI panel. Older transplant age and outpatient testing are risk factors for a positive GI panel. GI panel result is associated with changes to antibiotic therapy. Further characterization of differences in practitioner evaluation of acute diarrhea in solid organ transplant recipients, diarrheal etiologies in those with a negative GI panel, response to antibiotic therapy, and the impact of identified risk factors on graft rejection outcomes is needed.

## Data availability statement

The raw data supporting the conclusions of this article will be made available by the authors, without undue reservation.

## Ethics statement

The studies involving human participants were reviewed and approved by New York University Institutional Review Board [NYU IRB s18-01121 approval date 8/26/2018]. Written informed consent for participation was not required for this study in accordance with the national legislation and the institutional requirements.

## Author contributions

AV: formal analysis: lead; writing original draft: lead. AH: data curation: equal; project administration: lead; writing review and editing: supporting. AJ: data curation: equal; project administration: supporting. RM: data curation: supporting. BL: methodology: supporting; supervision: supporting; writing review and editing: supporting. JA: conceptualization: lead; methodology: supporting; supervision: lead; writing review and editing: lead. All authors contributed to the article and approved the submitted version.
