# Supplementary material for: Differences by transplant type in stool multiplex PCR testing for acute diarrhea in post-solid organ transplantation
Source: Front Gastroenterol (Lausanne). 2022 Dec 22;1:1064187. doi: 10.3389/fgstr.2022.1064187 (PMC12952319; doi:10.3389/fgstr.2022.1064187)
Supplement: Supplementary file 1 [file DataSheet_1.docx]

**Supplementary Table 1: Alternative Etiologies of Diarrhea in Patients with a Negative GI Panel at NYU Langone**

| **Diagnosis** | **Total Count (n=31)** | **Heart Transplant (n=2)** | **Liver Transplant (n=12)** | **Lung Transplant (n=2)** | **Renal Transplant (n=15)** |
| --- | --- | --- | --- | --- | --- |
| None Identified | 12 | 1 | 7 | 2 | 2 |
| Mycophenolate Mofetil Side Effect | 5 | 1 | 0 | 0 | 4 |
| Presumed Viral Gastroenteritis | 4 | 0 | 2 | 0 | 2 |
| CMV | 2 | 0 | 1 | 0 | 1 |
| Antibiotic-Associated Diarrhea | 2 | 0 | 0 | 0 | 2 |
| UTI/Pyelonephritis | 2 | 0 | 0 | 0 | 2 |
| Medication-Induced | 1 | 0 | 1 | 0 | 0 |
| Diet-Related | 1 | 0 | 0 | 0 | 1 |
| Overflow Diarrhea | 1 | 0 | 0 | 0 | 1 |
| Acute Bacterial Cholangitis | 1 | 0 | 1 | 0 | 0 |

**Supplementary Table 2: Adjusted Multivariable Regression Predicting Positive GI Panel**

|  | | | Outcome: Positive GI Panel | |
| --- | --- | --- | --- | --- |
| Variable | | | **Odds Ratio** | **95% Confidence Interval** |
| Age (years) | | | 0.995 | 0.978-1.01 |
| Female | | | 0.677 | 0.387-1.18 |
| **Non-White Race** | | | **2.58** | **1.44-4.70** |
| Charlson’s Comorbidity Index | | | 0.922 | 0.794-1.07 |
| OI Prophylaxis | | | 1.45 | 0.736-2.88 |
| Recent Antibiotics/Hospitalization | | | 1.02 | 0.561-1.88 |
| Transplant Age | | |  |  |
|  | 0-3 months | | **1** | **1** |
|  | **3-12 months** | | **5.56** | **1.59-23.3** |
|  | **12 months or older** | | **7.14** | **2.24-28.1** |
| Transplant Type | | |  |  |
|  | | Lung Transplant | 1 | 1 |
|  | | Heart Transplant | 2.41 | 0.982-6.04 |
|  | | Liver Transplant | 1.83 | 0.680-4.97 |
|  | | **Renal Transplant** | **2.98** | **1.27-7.16** |
| Immunosuppressive Medications | | |  |  |
|  | | None | 1.67 | 0.064-21.4 |
|  | | Calcineurin Inhibitors | 3.00 | 0.838-14.5 |
|  | | Mycophenolate Mofetil/Azathioprine | 1.51 | 0.762-3.02 |
|  | | Steroids | 1.40 | 0.699-2.84 |
|  | | **mTOR Inhibitors** | **0.291** | **0.071-0.973** |
| **Hospitalization** | | | **0.428** | **0.234-0.775** |
| Bold **= statistically significant** | | | |  |

**Supplementary Table 3: Pathogen and Therapy for Patients Receiving a Full Course of Empiric Antibiotic Therapy**

| Pathogen Identified | Number of Subjects (n=47) | Proportion of Subjects with Positive PCR Result Supporting Empiric Antibiotic Therapy Choice |
| --- | --- | --- |
| No Pathogen Identified | 35 | N/A |
| Norovirus GI/GII | 3 | 0 (0) |
| Rotavirus A | 1 | 0 (0) |
| Sapovirus (I, II, IV, and V) | 1 | 0 (0) |
| Enteropathogenic *E. coli* (EPEC) | 3 | 3 (100) |
| Enteroaggregative *E. coli* (EAEC) | 2 | 2 (100) |
| Campylobacter | 1 | 1 (100) |
| *C. difficile* Toxin A/B | 1 | 1 (100) |

**Supplementary Table 4: Baseline Characteristics by Transplant Type in Outpatient Subjects**

|  | | | **Evaluation by Transplant Type** | | | | | |
| --- | --- | --- | --- | --- | --- | --- | --- | --- |
|  | | | **Heart Transplant (n=14)** | | **Liver Transplant (n=21)** | **Lung Transplant (n=15)** | **Renal Transplant (n=46)** | **P-Value** |
| Age (years), median (IQR) | | | 59.7 (40.1-68.0) | 61.0 (39.0-66.3) | | 61.7 (53.7-69.0) | 53.9 (42.0-65.0) | 0.288 |
| Female | | | 7 (50.0) | 8 (38.1) | | 6 (40.0) | 20 (43.5) | 0.909 |
| Race | | |  |  | |  |  | 0.034 |
|  | | White | 9 (64.3) | 12 (57.1) | | 10 (66.7) | 15 (32.6) |  |
|  | | Non-White | 5 (35.7) | 9 (42.9) | | 5 (33.3) | 31 (67.4) |  |
| Ethnicity | | |  |  | |  |  | 0.753 |
|  | | Non-Hispanic | 11 (78.6) | 13 (61.9) | | 12 (80.0) | 32 (69.6) |  |
|  | | Hispanic | 3 (21.4) | 5 (23.8) | | 2 (13.3) | 8 (17.4) |  |
|  | | Other/Declined/Unknown | 0 (0) | 3 (14.3) | | 1 (6.7) | 6 (13.0) |  |
| Charlson’s Comorbidity Index, median (IQR) | | | 4.00 (3.00-5.00) | 4.00 (3.00-6.00) | | 3.00 (2.75-5.00) | 4.00 (3.00-6.00) | 0.406 |
| Human Immunodeficiency Virus (HIV), n (%) | | | 0 (0) | 2 (9.5) | | 0 (0) | 2 (4.3) | 0.428 |
| Sexual Exposure, n (%) | | | 0 (0) | 0 (0) | | 0 (0) | 2 (4.3) | 0.528 |
| Duration of Transplant, median (IQR) | | | 2390 (612-5270) | 1280 (245-2810) | | 643 (120-1580) | 711 (162-2360) | 0.463 |
| Transplant Age, n (%) | | |  |  | |  |  | 0.726 |
|  | | 0-3 months | 0 (0) | 2 (9.5) | | 1 (6.7) | 2 (4.3) |  |
|  | | 3-12 months | 2 (14.3) | 3 (14.3) | | 1 (6.7) | 9 (19.6) |  |
|  | | 12 months or older | 12 (85.7) | 17 (81.0) | | 13 (86.7) | 35 (76.1) |  |
| Immunosuppression, n (%) | | |  |  | |  |  |  |
|  | None | | 0 (0) | 0 (0) | | 0 (0) | 0 (0) | NA |
|  | Calcineurin Inhibitor | | 14 (100) | 16 (76.2) | | 15 (100) | 43 (93.5) | 0.024 |
|  | Mycophenolate Mofetil/Azathioprine | | 10 (71.4) | 10 (47.6) | | 13 (86.7) | 42 (91.3) | <0.001 |
|  | Steroids | | 10 (71.4) | 7 (33.3) | | 15 (100) | 32 (69.6) | <0.001 |
|  | mTOR Inhibitor | | 1 (7.1) | 7 (33.3) | | 1 (6.7) | 1 (2.2) | 0.001 |
|  | Other | | 0 (0) | 1 (4.8) | | 0 (0) | 1 (2.2) | 0.716 |
| Immunosuppression Held, n (%) | | | 4 (28.6) | 0 (0) | | 0 (0) | 7 (15.2) | 0.025 |
| Number of Immunosuppressive Agents, n (%) | | |  |  | |  |  | <0.001 |
|  | 0 | | 0 (0) | 0 (0) | | 0 (0) | 0 (0) |  |
|  | 1 | | 0 (0) | 8 (38.1) | | 0 (0) | 1 (2.2) |  |
|  | 2 | | 7 (50.0) | 7 (33.3) | | 1 (6.7) | 17 (37.0) |  |
|  | 3 | | 7 (50.0) | 5 (23.8) | | 14 (93.3) | 28 (60.9) |  |
|  | 4 | | 0 (0) | 1 (4.8) | | 0 (0) | 0 (0) |  |
| On Opportunistic Infection Prophylaxis | | | 3 (21.4) | 6 (28.6) | | 12 (80.0) | 23 (50.0) | 0.004 |
| Travel 30 Days Prior to PCR | | | 0 (0) | 2 (9.5) | | 0 (0) | 3 (6.5) | 0.467 |
| Recent Antibiotics or Hospitalization | | | 5 (35.7) | 7 (33.3) | | 5 (33.3) | 16 (34.8) | 0.999 |
| Symptoms at PCR | | |  |  | |  |  |  |
|  | | Hematochezia | 0 (0) | 0 (0) | | 0 (0) | 1 (2.2) | 0.778 |
|  | | Abdominal Pain | 2 (14.3) | 6 (28.6) | | 3 (20.0) | 12 (26.1) | 0.751 |
|  | | Fever | 1 (7.1) | 1 (4.8) | | 0 (0) | 4 (8.7) | 0.667 |
|  | | Nausea/Vomiting | 1 (7.1) | 1 (4.8) | | 3 (20.0) | 9 (19.6) | 0.318 |
|  | | Other/Unknown | 1 (7.1) | 1 (4.8) | | 1 (6.7) | 0 (0) | 0.386 |

**Supplementary Table 5: GI Panel Results by Transplant Type in Outpatient Subjects**

|  | | |  | **Evaluation By Transplant Type** | | | |
| --- | --- | --- | --- | --- | --- | --- | --- |
|  | | | **Heart Transplant (n=14)** | **Liver Transplant (n=21)** | **Lung Transplant (n=15)** | **Renal Transplant (n=46)** | **P-Value** |
| Positive GI PCR Panel | | | 7 (50.0) | 8 (38.1) | 7 (46.7) | 29 (63.0) | 0.258 |
| Viral infection, n (%) | | | 3 (21.4) | 2 (9.5) | 5 (33.3) | 16 (34.8) | 0.159 |
| Bacterial Infection, n (%) | | | 4 (28.6) | 6 (28.6) | 1 (6.7) | 17 (37.0) | 0.169 |
| Parasitic Infection, n (%) | | | 0 (0) | 0 (0) | 1 (6.7) | 3 (6.5) | 0.495 |
| Multiple Pathogens | | | 3 (21.4) | 1 (4.8) | 1 (6.7) | 8 (17.4) | 0.503 |
| Viral Infection | | |  |  |  |  |  |
|  | | Adenovirus | 0 (0) | 0 (0) | 0 (0) | 0 (0) | NA |
|  | | Astrovirus | 0 (0) | 0 (0) | 0 (0) | 1 (2.2) | 0.778 |
|  | | Norovirus | 2 (14.3) | 2 (9.5) | 2 (13.3) | 12 (26.1) | 0.352 |
|  | | Rotavirus | 0 (0) | 0 (0) | 2 (13.3) | 0 (0) | 0.012 |
|  | | Sapovirus | 1 (7.1) | 0 (0) | 2 (13.3) | 2 (4.3) | 0.343 |
| Bacterial Infection | | |  |  |  |  |  |
|  | | *Campylobacter* species | 2 (14.3) | 0 (0) | 0 (0) | 4 (8.7) | 0.220 |
|  | | *Clostridioides difficile* | 5 (35.7) | 0 (0) | 3 (20.0) | 8 (17.4) | 0.046 |
|  | | *Plesiomonas shigelloides* | 0 (0) | 0 (0) | 0 (0) | 0 (0) | NA |
|  | | *Salmonella* species | 0 (0) | 0 (0) | 0 (0) | 0 (0) | NA |
|  | | *Yersinia enterocolitica* | 0 (0) | 1 (4.8) | 0 (0) | 0 (0) | 0.307 |
|  | | *Vibrio parahaemolyticus* | 0 (0) | 0 (0) | 0 (0) | 0 (0) | NA |
|  | | *Vibrio vulnificus* | 0 (0) | 0 (0) | 0 (0) | 0 (0) | NA |
|  | | *Vibrio cholerae* | 0 (0) | 0 (0) | 0 (0) | 0 (0) | NA |
|  | | *Enteroaggregative E. coli (EAEC)* | 0 (0) | 1 (4.8) | 0 (0) | 4 (8.7) | 0.434 |
|  | | *Enteropathogenic E. coli (EPEC)* | 3 (21.4) | 3 (14.3) | 0 (0) | 9 (1`9.6) | 0.295 |
|  | | *Enterotoxigenic E. coli (ETEC)* | 1 (7.1) | 0 (0) | 0 (0) | 1 (2.2) | 0.471 |
|  | | *Shiga-like Toxin-producing E. coli (STEC)* | 0 (0) | 1 (4.8) | 0 (0) | 0 (0) | 0.307 |
|  | | *E. coli O157* | 0 (0) | 0 (0) | 0 (0) | 0 (0) | NA |
|  | | *Shigella/Enteroinvasive E. coli (EIEC)* | 0 (0) | 1 (4.8) | 0 (0) | 0 (0) | 0.307 |
| Parasitic Infection | | |  |  |  |  |  |
|  | | *Cryptosporidium* | 0 (0) | 0 (0) | 0 (0) | 1 (2.2) | 0.778 |
|  | | *Cyclospora cayatenesis* | 0 (0) | 0 (0) | 0 (0) | 0 (0) | NA |
|  | | *Entamoeba histolytica* | 0 (0) | 0 (0) | 0 (0) | 0 (0) | NA |
|  | | *Giardia lamblia* | 0 (0) | 0 (0) | 2 (13.3) | 1 (2.2) | 0.092 |
| Antibiotics Prescribed | | | 6 (42.9) | 6 (28.6) | 5 (33.3) | 25 (54.3) | 0.193 |
|  | Incomplete Empiric Course Before GI Panel Result | | 0 (0) | 0 (0) | 0 (0) | 1/25 (4.0) | 0.778 |
|  | Empiric Antibiotics Narrowed After GI Panel Result | | 1/6 (16.7) | 0 (0) | 0 (0) | 2/25 (8.0) | 0.545 |
|  | Directed Antibiotics Initiated After GI Panel Result | | 5/6 (83.3) | 3/6 (50.0) | 3/5 (60.0) | 19/25 (76.0) | 0.112 |
|  | Full Empiric Course Unchanged After GI Panel Result | | 0 (0) | 3/6 (50.0) | 2/5 (40.0) | 3/25 (12.0) | 0.402 |
| Antibiotic Management Affected by GI Panel Result | | |  |  |  |  |  |
| Hospitalization Within 30 Days of PCR | | | 1 (7.1) | 0 (0) | 3 (20.0) | 7 (15.2) | 0.196 |
| ER Visit Within 30 Days of PCR | | | 2 (14.3) | 2 (9.5) | 1 (6.7) | 1 (2.2) | 0.357 |
| Surgery Within 30 Days of PCR | | | 0 (0) | 1 (4.8) | 1 (6.7) | 1 (2.2) | 0.706 |
| Death Within 30 Days of PCR | | | 0 (0) | 0 (0) | 1 (6.7) | 3 (6.5) | 0.495 |
| Endoscopy Within 30 Days of PCR | | | 1 (7.1) | 3 (14.3) | 0 (0) | 2 (4.3) | 0.306 |
